# 25-Hydroxycholecalciferol Inhibits Cell Growth and Induces Apoptosis in SiHa Cervical Cells via Autocrine Vitamin D Metabolism

**DOI:** 10.3390/biomedicines11030871

**Published:** 2023-03-13

**Authors:** Rivak Punchoo, Greta Dreyer, Tahir S. Pillay

**Affiliations:** 1 Tshwane Academic Division, National Health Laboratory Service (NHLS), Pretoria 0001, South Africa; 2 Department of Chemical Pathology, Faculty of Health Sciences, University of Pretoria, Pretoria 0001, South Africa; 3 Department of Obstetrics and Gynaecology, Faculty of Health Sciences, University of Pretoria, Pretoria 0001, South Africa; 4 Division of Chemical Pathology, Department of Pathology, University of Cape Town, Rondebosch 7701, South Africa

**Keywords:** cervical cancer, SiHa, 25-hydroxycholecalciferol, apoptosis, vitamin D metabolising system, 24-hydroxylase (CYP24A1), 1-alpha hydroxylase (CYP27B1), vitamin D receptor (VDR)

## Abstract

Preclinical studies show that the anticancer actions of vitamin D metabolites are mediated by apoptosis, inhibition of cell proliferation and induction of cell cycle arrest. Cervical cancer cells express an autocrine vitamin D metabolising system (VDMS) comprised of a vitamin D receptor, vitamin D catabolic enzyme (CYP24A1), and the activating enzyme of 25-hydroxycholecalciferol (25(OH)D3), CYP27B1. We assessed the anticancer effects of 25(OH)D3 at clinically relevant concentrations on a cervical squamous cell cancer cell line, SiHa. We evaluated cell health parameters (cell count, viability, and cell cycle), cell death modes (apoptosis, autophagic-dependent death, and necrosis by flow cytometry and transmission electron microscopy), and autocrine VDMS gene and protein expression by qPCR and Western blot, respectively. Our study demonstrates that physiological and supraphysiological doses of 25(OH)D3 inhibit cell growth and viability and induce biochemical and morphological apoptosis in SiHa cells. These growth effects are mediated by alteration in the VDMS gene and protein expression, with prominent negative feedback at supraphysiological treatment dose. These data identify promising therapeutic potential of 25(OH)D3 in cervical cancer, which warrants further clinical translational investigations.

## 1. Introduction

Cancer of the uterine cervix is the fourth most common cancer in women worldwide, with a high incidence and prevalence in resource-constrained settings. The oncogenic subtypes of human papilloma virus (HPV) are detected in 99.7% of cervical cancers [1]. HPV disrupts the normal cell cycle and promotes the accumulation of genetic damage. Persistent viral infection of the uterine cervical epithelium causes dysplasia and the development of precancerous lesions, which can progress to carcinoma and metastasise [1,2,3]. Cervical cancer imposes significant morbidity, mortality, and health expenditure [4]. For example, in Central America and Africa, it causes the highest cancer-related mortality among women [5,6]. Cervical cancer is treated with surgery, chemotherapy, and radiotherapy [7,8]. Chemotherapy and adjunctive nutritional support, accessible and affordable in a resource-constrained environment, may enhance positive treatment outcomes [9].

Preclinical studies have shown variable evidence for the anticancer effects of vitamin D metabolites, with the most robust evidence in colorectal, breast, and prostate cancers [10,11,12,13,14]. The most frequent molecular mechanisms of the anticancer actions of the vitamin D metabolome are attributed to the inhibition of cell proliferation, arrest of cell cycling, induction of apoptosis and autophagy, and inhibition of angiogenesis in tumours [10,15].

The systemic metabolism of vitamin D is well described in humans [16]. Vitamin D (cholecalciferol; D_3_) is synthesised in the skin under the action of UVB light on the skin precursor 7-dehydrocholesterol or obtained from dietary sources such as dairy, liver, and fish. In contrast, ergocalciferol (D_2_) is obtained from dietary products found in, for example, yeast and mushrooms. D_2_ and D_3_ forms are hydroxylated in the liver by activating cytochrome P450 (CYP) mixed-function oxygenase enzymes in mitochondria or microsomes. The 25-hydroxylases (most commonly, CYP27A1, CYP2R1, CYP2J2/3, CYP3A4, CYP2D25, and CYP2C11) hydroxylate D_2_ and D_3_ to form 25-hydroxycholecalciferol (25(OH)D3 or 25(OH)D_3_) [17]. In the kidney, CYP27B1 hydroxylates 25(OH)D3 to produce 1,25 dihydroxycholecalciferol (1,25(OH)D3). 25(OH)D3 and 1,25(OH)D3 are biologically active hormones that CYP24A1 degrades in the kidney by a negative feedback mechanism, forming excretory and inactive products or partial agonists to maintain cellular homeostasis in target tissues. The activated forms, especially 1,25(OH)D3, transduce intracellular signals by heterodimerising with vitamin D receptor (VDR) and co-receptor RXR. The heterodimer binds to vitamin D response elements (VDREs) in multiple regulatory regions at promoters, distal sites of vitamin-D-responsive genes responsible for cell health and recruits co-modulators of gene expression. Non-genomic actions of 1,25(OH)D3 have also been described, which effect rapid intracellular actions. A wide array of VDR target genes have been identified in 1,25(OH)D3’s anticancer actions [10,16,18]. Thus, using vitamin D metabolites and exploiting systemic, autocrine, and paracrine vitamin D metabolism in regulating cancer pathways are potentially attractive approaches for treating cancers.

The extrarenal autocrine and paracrine vitamin D system synthesises, degrades, and transduces vitamin D signals in some organ systems that are necessary to maintain healthy cell growth and counteract mutagenic stimuli [19]. Uterine cervical cells express an autocrine vitamin D metabolising system (VDMS) consisting of the cytochrome P450 family of activating enzymes and a catabolic enzyme (CYP24A1). The VDMS signals via vitamin D receptor (VDR) transcription factor to control the expression of various genes, including genes regulating cell growth and cell death. The fact that the VDMS is expressed in only a few tissues in the human body suggests that autocrine homeostasis of vitamin D metabolism is important to optimise cellular homeostasis and, more broadly support the traditional systemic metabolism of vitamin D via the liver and kidney [20,21]. The cell line SiHa is a squamous cell carcinoma from primary uterine cervical tissue obtained from a 55-year-old female Japanese patient [22], and it is calcitriol-responsive, as it contains VDR and activating and inactivating vitamin D enzymes [22,23].

There is limited evidence for the anticancer effect of vitamin D metabolites in cervical cancer, with limited exploration of dose-dependent effects of precursor vitamin D metabolite 25(OH)D3 on cell growth and modes of cell death. In addition, the regulation of the VDMS in cervical cancer by 25(OH)D3 is poorly understood. Therefore, this study aimed to identify the dose–response anticancer effects of 25(OH)D3 at both physiological and supraphysiological levels in SiHa cells. This cervical cancer cell line represents the most prevalent histological type, i.e., squamous cell cervical cancer [7]. The anticancer parameters investigated included cell health indices (cell viability, cell proliferation, and cell cycle) and modes of cell death (apoptosis, autophagy, and necrosis). Furthermore, the gene and protein regulation of the autocrine SiHa VDMS was assessed by 25(OH)D3 treatments. We show that physiological and supraphysiological doses of 25(OH)D3 inhibit cell growth and viability and induce biochemical and morphological apoptosis in SiHa cells. These growth effects are mediated by alteration in the VDMS gene and protein expression of synthetic and catabolic enzymes and the VDR, with prominent negative feedback at a supraphysiological treatment dose in SiHa cells.

## 2. Materials and Methods

### 2.1. Culture of Control and Experimental SiHa Cells and Overview of Experimental Design

The SiHa cell line (HTB-35™) was obtained from the American Type Culture Collection (ATCC, Manassas, VA, USA) and grown in Basal Medium Eagle (BME) (supplemented with 10% foetal calf serum (Thermo Fisher Scientific; Waltham, MA, USA), 100 μg mL^−1^ streptomycin, 100 U/mL penicillin G, and 250 μg/L fungizone) in a humidified incubator at 5% CO_2_ and 37 °C. Cells were treated with a dose range of 25-hydroxycholecalciferol that was informed by clinical guidelines for serum 25(OH)D3 levels divided into four categories: 25 nM (deficient), 60 nM (insufficient), 250 nM (physiological), and 2500 nM (supraphysiological) doses [24]. The 25-hydroxycholecalciferol (Sigma-Aldrich, St Louis, MI, USA) doses were prepared in analytical-grade ethanol (Sigma-Aldrich; St Louis, MI, USA).

Exponentially growing SiHa cells were seeded at 25,000 cells/mL in 25 cm^2^ flasks or 5000 cells/well in 96-well plates, and control and experimental cells were harvested at 72 h. The solvent control used the diluent ethanol diluted in culture medium to a concentration corresponding to the supraphysiological treatment and to a final concentration of 0.1% (*v*/*v*) ethanol. Positive control cultures were treated with rapamycin (for the autophagy-dependent death assay) and actinomycin (apoptosis assays) to a final concentration of 200 nM and 0.1 μg/mL, respectively. All assays were conducted in three independent experiments, and each experiment was conducted in triplicate.

Cell health parameters were analysed by cell enumeration using a crystal violet assay, cell viability was assessed using an MTT assay, cell proliferation was assessed by a Ki67 flow cytometric assay, and cell cycle was assessed by flow cytometry. Modes of cell death were analysed by early and late apoptosis (mitochondrial membrane potential, Annexin V assay, and caspase 3/7 flow cytometric assays), autophagy (LC3-II flow cytometric assay), and necrosis (LDH release assay). Morphological features of cell death were assessed by transmission electron microscopy. The gene and protein expressions of the VDMS were analysed by qPCR and Western blotting, respectively.

### 2.2. Crystal Violet Assay

The crystal violet colorimetric assay quantifies cell biomass in adherent cell culture. It is based on crystal violet triarylmethane dye binding to intracellular molecules such as DNA and protein. Colour intensity is proportional to the cell biomass, and the solubilised dye is quantified by absorbance at 570 nm [25].

Glutaraldehyde (#111308) and Triton X-100 (#9002931) were purchased from Sigma-Aldrich (St Louis, MI, USA), and crystal violet staining powder was purchased from Merck (Darmstadt, Germany). SiHa control and experimental cells were counted following a standardised protocol [25]. At 72 h, the samples were washed with 1X PBS, fixed with 100 μL of 1% glutaraldehyde, and stained with 100 μL of 0.1% crystal violet for 30 min. Thereafter, cells were washed and dried, the dye was solubilised using 200 μL 0.2% Triton X-100 and incubated for 30 min, 100 μL of the sample was transferred to a sterile 96-well plate, and the absorbance was quantified on an ELx800 universal microplate reader (Bio-Tek Instruments Inc., Winooski, VT, USA). All absorbances were blank-corrected, and the percentage cell count was normalised to solvent control and expressed as a percentage.

### 2.3. 3-(4,5-Dimethylthiazol-2-yl)-2,5-Diphenyltetrazolium Bromide (MTT) Viability Assay

MTT is a colorimetric assay that quantifies cell viability based on metabolic function. The water-soluble MTT reagent is converted to an insoluble formazan by viable mitochondria. The formazan is solubilised, and its concentration is determined by absorbance at 570 nm [26].

The cell viability of control and experimental SiHa cultures was evaluated by a standardised protocol [26]. In brief, after 72 h incubation, the control and experimental cultures were washed with 1X PBS, and 100 μL of 5 mg/mL MTT reagent (Sigma-Aldrich, St. Louis, MI, USA) dissolved in RPMI (Roswell Park Memorial Institute Medium) without phenol red (Sigma-Aldrich, St. Louis, MI, USA) was added, followed by incubation for 4 h under standard conditions. Next, 100 μL of the solubilising solution (isopropanol, 0.1 N HCl, 10% Triton X-100) (Sigma-Aldrich, St. Louis, Missouri, MI, USA) was added, and the plate covered in foil and incubated at room temperature overnight or until purple crystals were dissolved. Then, absorbance was measured at 570 nm, and the percentage viability was calculated [26].

### 2.4. Cell Proliferation Ki67 Flow Cytometric Assay

Cell proliferation can be measured by increased Ki67 nuclear antigen in proliferating cells [27].

A Muse^TM^ Ki67 flow cytometric proliferation kit (#MCH100114) containing fixation solution, assay buffer, permeabilisation solution, and Muse^TM^ Hu Ki67-PE was purchased from Merck (Darmstadt, Germany). Siha cells harvested at 72 h were stained following the manufacturer’s instructions [28]; cells were fixed with 1X fixation solution and washed with 1X assay buffer, and each sample was permeabilised with 100 μL fixation buffer containing fluorescently labelled Ki67 antibody. The samples were incubated for 30 min and analysed with a Muse™ cell analyser (Merck, Darmstadt, Germany) as described by the manufacturer’s protocol [28].

### 2.5. Cell Cycle Analysis by Flow Cytometry

The cell cycle is critical to cell survival, and disruption of mitotic cell division frequently occurs in cancer cells.

The Muse™ cell cycle assay purchased from Merck (Darmstadt, Germany) uses a DNA intercalating stain to identify cells in the cell cycle’s G0/G1, S, and G2/M phases on the Muse™ cell analyser. The manufacturer’s standardised protocol and reagents were used to perform the assay [29]. Control and experimental cell suspensions (200 μL) were centrifuged and washed with 1X PBS, then fixed using 200 μL of ice-cold ethanol and incubated for 180 min at −20 °C. The cells were then centrifuged at 300× *g* for 5 min, washed once with 1X PBS, stained with 200 μL of Muse™ cell cycle reagent, incubated for 30 min protected from light, and analysed on a Muse™ cell analyser (Merck, Darmstadt, Germany).

### 2.6. Mitochondrial Membrane Depolarisation Flow Cytometric Assay

Mitochondrial transmembrane potential (∆Ψm) enables cells to synthesise ATP, and depolarisation of ∆Ψm is a common biomarker of early apoptosis, providing a reliable indicator of mitochondrial dysfunction and ill cell health [30].

The Muse™ Mitopotential kit purchased from Merck (Darmstadt, Germany) identifies cells with depolarised mitochondrial potential from live and dead cell populations. The manufacturer’s standardised protocol and reagents were used to perform the assay [30]. SiHa cells were harvested at 72 h using trypsin and resuspended in 1X PBS solution. The positive control was actinomycin D (0.1 μg/mL). In brief, 95 μL of mitopotential working solution was added to 100 μL of cell suspension and incubated under standard cell culture conditions for 20 min. Thereafter, 5 μL of 7-ADD was added and incubated for 5 min at room temperature. The suspension was mixed thoroughly, measured, and analysed on a Muse™ cell analyser (Merck, Darmstadt, Germany).

### 2.7. Annexin V Flow Cytometric Assay

Apoptosis is characterised by a flipped pattern of phosphatidylserine (PS) residues, which locates PS residues from the inner cell membrane to the external cell membrane surface [31]. Annexin V binds externalised PS and indicates late apoptosis [32]. The use of an additional cell death flow cytometric biomarker in this assay differentiates cells that have permeabilised cell membranes from cells with intact membranes that exclude entry of the dead cell biomarker (i.e., live and early apoptosis populations) [33].

SiHa control and experimental cells were seeded as described above for 72 h, then prepared for the Annexin V assay using the manufacturer’s reagent and protocol [34]. The positive control culture was treated with actinomycin D to a final concentration of 0.1 μg/mL. In brief, cells were washed in 1X PBS, and 50 μL of cell suspension was added to 5 μL of antibody reagent and incubated under standard cell culture conditions for 30 min. Thereafter, 150 μL of dead cell marker was added, mixed thoroughly, and measured and analysed on a Muse™ cell analyser (Merck, Darmstadt, Germany).

### 2.8. Caspase 3/7 Flow Cytometric Detection Assay

Cysteinyl-directed, aspartate-specific proteases (caspases) are a family of cysteine-dependent endoproteases that hydrolyse their substrates after specific aspartic acid residues [35]. Late apoptosis is characterised by the activation of terminal caspases such as caspase-3 and caspase-7, which are responsible for the morphological changes evidenced in advanced apoptotic cells [36].

A Muse™ caspase 3/7 kit containing caspase 3/7 working solution and 7-ADD dead cell marker was purchased from Merck (Darmstadt, Germany). In brief, SiHa cells were seeded as described above in Section 2.1 for 72 h. The positive control culture was treated with actinomycin D to a final concentration of 0.1 μg/mL. Cells were prepared, measured, and analysed using the manufacturer’s reagent and protocol [37]. Samples were analysed by adding 5 μL of caspase 3/7 working solution to 50 μL of cell suspension and incubated under standard cell culture conditions for 30 min. Then, 150 μL of dead cell marker was added, and the sample was mixed thoroughly. Caspase 3/7 detection was measured and analysed on a Muse™ cell analyser (Merck, Darmstadt, Germany) as prescribed by the manufacturer [37].

### 2.9. Autophagic Cell Death Flow Cytometric Assay

Autophagy-dependent cell death is a form of regulated cell death characterised by vacuolisation, the formation of a double-membrane structure, the autophagosome, and digestion and subsequent recycling of cytoplasmic contents and organelles by the lysosome. To identify autophagic-dependent cell death, autophagosomes can be investigated by TEM, and autophagy-related proteins such as LC3-II (lipidated form) can be quantified [38].

The control and experimental SiHa cells were harvested at 72 h and prepared and analysed using the manufacturer’s reagents and instructions [39]. The positive control culture was treated for six hours with 200 nM rapamycin (Sigma-Aldrich, St Louis, MI, USA). In brief, harvested cells were centrifuged at 300× *g* for 5 min at 4 °C, and the supernatant was removed, then stained with 5 μL anti-LC3 antibody and 95 μL of 1X assay buffer for 30 min on ice. Cells were re-spun at 300× *g* for 5 min at 4 °C; then, the supernatant was removed, and cells were washed with 1X assay buffer and resuspended in 200 μL 1X assay buffer. Measurement and analysis were performed on a Muse™ cell analyser (Merck, Darmstadt, Germany), which quantified LC3-II-positive cells.

### 2.10. Lactate Dehydrogenase (LDH) Release Assay

The detection of LDH released from cultured cells with permeabilised cell membranes into the culture medium provides a biochemical measure of cytotoxicity and cell necrosis characterised by cardinal permeabilised cell membranes [40].

SiHa control and experimental cultures at 72 h were assessed by a manual LDH release assay by a standardised protocol [40], which quantifies LDH activity using a colorimetric endpoint assay. Briefly, the maximum LDH release wells and volume correction control wells were incubated for 45 min. Thereafter, 50 μL of the supernatant from each of the wells was transferred to a sterile 96-well plate, and 50 μL of the 2X LDH assay buffer was added to the supernatant. The reagent was mixed gently by shaking the plates for 30 s and incubated at room temperature for 30 min covered in foil. Then, 50 μL acetic acid stop reagent was added, and the plate was shaken for 30 s to mix the reagent. The absorbance was measured at 490 nm using an ELx800 universal microplate reader (Bio-Tek Instruments Inc, Winooski, VT, USA). The percentage (%) of cytotoxicity was calculated for control and experimental cultures and corrected for absorbance in the untreated well and relative to the maximum LDH release well, as described in the protocol [40].

### 2.11. Transmission Electron Microscopy (TEM)

Ultrastructural analyses by TEM of control and experimental cultures provided data on cellular characteristics of cell death.

SiHa cells were harvested by trypsin and washed with 1X PBS. SiHa cells were fixed in 2.5% glutaraldehyde in 0.075 M phosphate buffer for 1 h. Thereafter, cells were rinsed three times with 0.075 M phosphate buffer and fixed with osmium tetroxide for 30 min. Cells were dehydrated with a graded ethanol series (30%, 50%, 70%, 90%, and 100%). Subsequently, cells were infiltrated with 50% Quetol in 100% ethanol (1:1) for 1 h, then with 100% Quetol for 6 h. Ultra-thin sections were prepared using an ultramicrotome, and sections were placed onto copper support grids, which were then contrasted with 4% uranyl acetate and counterstained with lead citrate for 10 min each. Micrographs were captured on a JOEL JEM 2100F (JOEL Ltd., Tokyo, Japan) transmission electron microscope [41]. Micrographs were qualitatively assessed for ultrastructural abnormalities, particularly features that characterise apoptosis, autophagy-dependent death, and necrosis [36].

### 2.12. Reverse Transcription-Quantitative Polymerase Chain Reaction (qPCR)

qPCR was used to analyse the relative quantification of messenger RNA (mRNA) transcript expression between control and experimental cultures. The genes of interest were amplified and monitored in real-time by PCR using the cDNA template [42].

SiHa control and experimental cultures were incubated for 72 h. The total RNA was extracted with Qiazol lysis reagent (Qiagen Inc., Hilden, Germany). Briefly, RNA concentration was measured with a Nanodrop 2000 instrument (Thermo Fisher, Waltham, MA, USA) and standardised to 200 ng/μL with nuclease-free water. cDNA was synthesised using iScript reverse transcriptase supermix (Bio-Rad, Hercules, CA, USA) with the following thermal profile: priming, 5 min at 25 °C; reverse transcription, 20 min at 46 °C; and reverse transcriptase inactivation, 1 min at 95 °C on a Rotor-Gene Q thermal cycler (Germantown, MD, USA). The PCR reactions were performed using published primer sets [43], and reactions were amplified on a Qiagen Rotor-Gene Q 5Plex PCR thermocycler (Qiagen, Hilden, Germany) using iTaq Universal SYBR Green Supermix (Bio-Rad, Hercules, CA, USA). The thermal cycling profile for optimisation reactions was as follows: denaturation at 95 °C for 15 s, with a primer-specific annealing temperature for 15 s, and extension at 72 °C for 10 s for 40 cycles. Relative gene expression was determined using the delta delta Ct method [44]. The reference 18s rRNA gene was used as an internal control, and all Ct values were presented relative to the solvent control.

### 2.13. Western Blots of the VDMS

Immunoblotting of proteins of interest by Western blots was performed to analyse the VDMS relative protein expression. The total protein cell extracts were separated by gel electrophoresis and transferred to transfer membranes, which were then probed using antibodies targeting proteins of interest and semi-quantified by densitometric analysis [45].

Total protein was extracted from the SiHa control and experimental cultures after 72 h incubation using 500 μL MPER mammalian protein extraction buffer supplemented with protease inhibitors (Halt™ Protease Inhibitor Cocktail, EDTA-Free; 1:100). Protein concentration was determined using a Pierce™ BCA protein assay kit (Thermo Fisher, Waltham, MA, USA), and absorbance was read at 562 nm using an ELx800 absorbance reader. Protein samples (20 µg) were loaded onto NuPAGE™ 4–12% Bis-Tris protein gels, and SDS-PAGE was performed at 200 V for 50 min at room temperature in 1X MOPS running buffer (Bio-Rad, Hercules, CA, USA). Separated proteins were transferred to polyvinylidene fluoride (PVDF) membranes for 90 min at 110 V at 4 °C. PVDF membranes were blocked with 2.5% BSA in 0.2% PBS-Tween^®^ 20 for 1 h at room temperature. The membranes were then probed with the primary antibody cocktails against CYP27B1 (1:500), VDR (1:500), and CYP24A1 (1:1000) overnight at 4 °C. Membranes were washed, then probed with secondary antibody for 1 h at room temperature. Membranes were probed with HRP-conjugated anti-β-actin antibody in 0.2% PBS-Tween^®^ 20. β-actin housekeeper protein was used as a loading control. For reprobing, membranes were incubated in mild stripping buffer (1.5% glycine, 0.5% SDS, 1% Tween 20, pH 2.2) for 1 h. Membranes were developed using Pierce™ enhanced chemiluminescent reagent, and images were visualised and electronically photographed by a ChemiDoc™ XRS+ imaging system (Bio-Rad Laboratories Inc., Hercules, CA, USA). Protein blots were densitometrically analysed using Image Lab v6.0 software (Bio-Rad Laboratories, Inc., Hercules, CA, USA). The VDMS protein quantities were normalised to the corresponding β-actin expression for each gel. Semi-quantitative analysis was conducted on each of the three independent experiments for each of the blotted VDMS proteins.

### 2.14. Statistical Analyses

Statistical analysis was performed using three independent experiments, with each experiment performed in triplicate. The results were expressed as mean ± standard errors of the mean (SEM). Statistical analyses were performed using a one-way analysis of variance (ANOVA) and Bonferroni’s *post hoc* test using GraphPad Prism (La Jolla, CA, USA) statistical software; *p* ≤ 0.05 was considered significant.

## 3. Results

### 3.1. Assessment of Cell Growth Parameters by Enumerating Cell Count, Cell Viability, Cell Proliferation, and Cell Cycle in SiHa Cervical Cells Treated with 25(OH)D3

#### 3.1.1. 25(OH)D3 Significantly Reduced Cell Count and Viability in Both Physiological (250 nM) and Supraphysiological (2500 nM) Treatments but Did Not Affect Cell Proliferation in SiHa Cells

**Figure 1:**
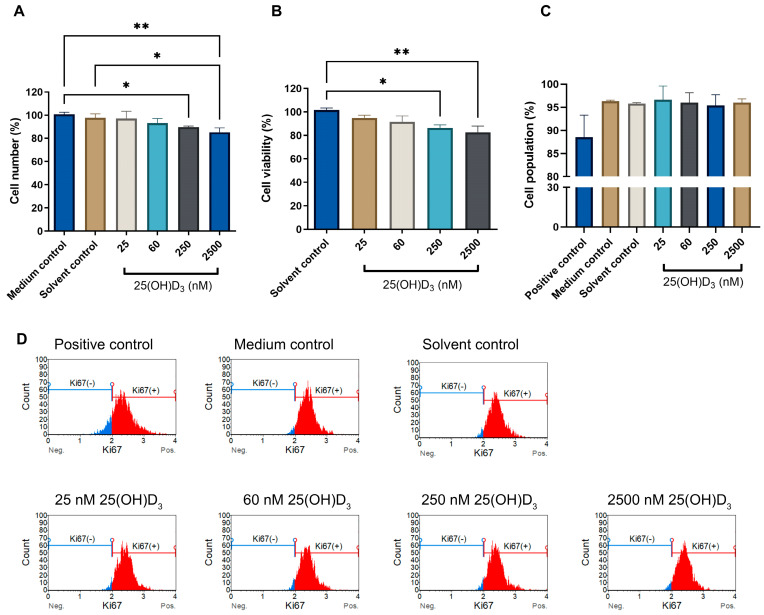
The effect of 25(OH)D3 on cell count (**A**), cell viability (**B**), and cell proliferation (**C**) in SiHa cells. Cell proliferation flow cytometry histograms (**D**) of control and experimental cultures stained for Ki67, show Ki67-positive cell populations (red) and Ki67negative populations (blue). The positive control was treated with actinomycin to a final concentration of 0.1 μg/mL. Data are expressed as mean (±SEM) from three independent experiments, each performed in triplicate (* *p* ≤ 0.05; ** *p* ≤ 0.01).

Analysis of cell health parameters identified a significant percentage reduction in both cell count and cell viability in both physiological (*p* < 0.05) and supraphysiological (*p* < 0.01) treatments (Figure 1). The Ki67 nuclear protein marker detected actively proliferating cells but did not show significant change (Figure 1). Collectively, these data indicate that 250 nM and 2500 nM 25(OH)D3 treatments inhibited cell count and viability but did not affect cell proliferation in SiHa cervical cells.

#### 3.1.2. 25(OH)D3 Significantly Increased the Sub-G1 Phase without Inducing Cell Cycle Arrest

**Figure 2:**
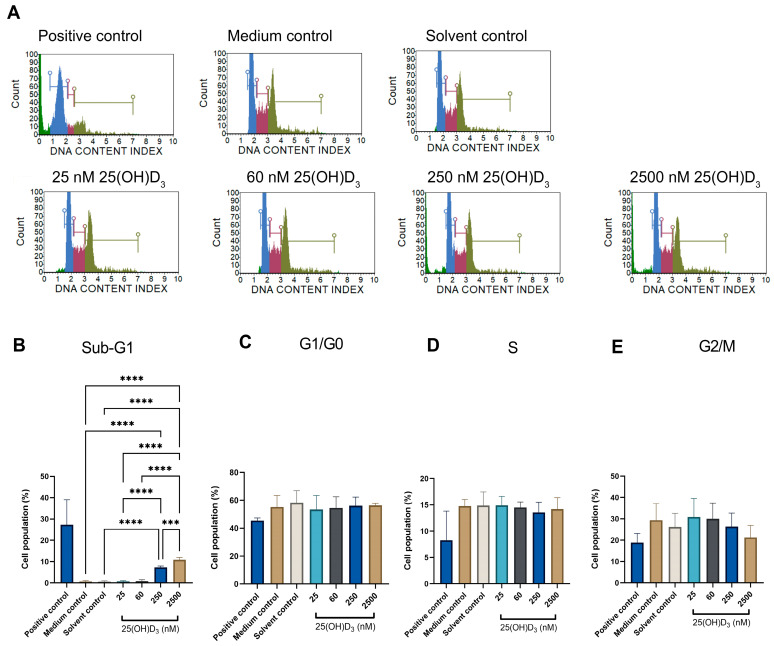
Cell cycle analysis by flow cytometry showing cell cycle histograms of control and experimental SiHa cells (**A**), and statistical analysis of Sub-G1, G1/G0, S and G2/M cell phases ((**B**–**E**), respectively)). The positive control was treated with actinomycin to a final concentration of 0.1 μg/mL. Data are expressed as mean (±SEM) from three independent experiments, each performed in triplicate (*** *p* ≤ 0.001, **** *p* ≤ 0.0001).

The cell cycle progression analysed by the Muse™ cell cycle kit measures the accumulation of DNA in the G0/G1, S and G2/M phases of the cell cycle [29]. No growth arrest was observed in either the G1 or G0 phase at all treatment doses, indicating that cells progressed through all cell phases without arrest (Figure 2). However, significant accumulation of cells in the sub-G1 phase was evident in physiological (7.30 ± 0.360) and supraphysiological (10.87 ± 0.617) treatments compared to solvent (0.567 ± 0.260). The propidium iodide fluorochrome in flow cytometry binds and labels DNA content, enabling a precise evaluation of cellular DNA content and subsequent identification of hypodiploid cells [46]. The elevated Sub-G1 population is consistent with an elevated dead cell population and cells containing nuclear fragmentation and low-molecular-weight DNA (fractional DNA content) [47,48,49]. In addition, the increased Sub-G1 phase correlates with the reduced cell count and cell viability observed in Figure 1. Furthermore, this finding is corroborated by the absence of significant changes in Ki67 expression, indicating that the cells mitotically divided, cycled through all cell cycle phases, and avoided cell cycle arrest events in both the G1 and G2 phases. Therefore, 25(OH)D3 does not exert an antiproliferative action or disrupt the cell cycle in SiHa cells.

### 3.2. Physiological and Supraphysiological Treatments with 25(OH)D3 in SiHa Cells Caused Upregulation of Early and Late Apoptotic Biochemical Biomarkers and Morphological Apoptosis; However, Modes of Autophagic Cell Death and Necrosis Were Absent

#### 3.2.1. Physiological and Supraphysiological Treatment with 25(OH)D3 Caused Significant Disruption of the Mitochondrial Transmembrane Potential Gradient

**Figure 3:**
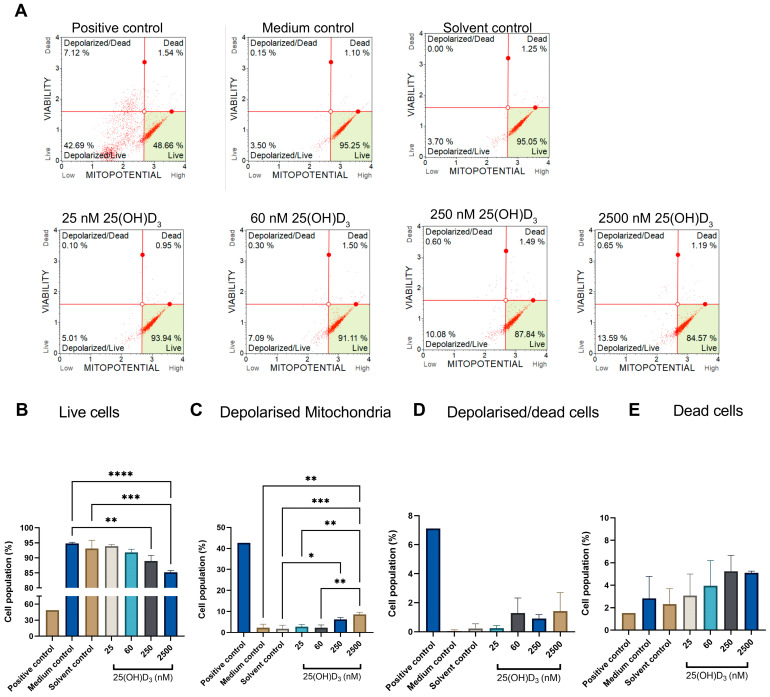
The effect of 25(OH)D3 on mitochondrial transmembrane potential (∆Ψm) in SiHa cells was assessed by flow cytometry. Each dot plot of SiHa cells is divided into four stages: live, depolarised/live, depolarised/dead, and dead cell populations (**A**). Analysis of the changes in ∆Ψm across the four stages of mitochondrial depolarisation is represented by bar graphs (**B**–**E**). The positive control was treated with actinomycin to a final concentration of 0.1 μg/mL. Data are expressed as mean (±SEM) from three independent experiments, each performed in triplicate (* *p* ≤ 0.05; ** *p* ≤ 0.01; *** *p* ≤ 0.001, **** *p* ≤ 0.0001).

Siha cells demonstrated a significant increase in the cell population with depolarised mitochondrial transmembrane potential at physiological treatment (6.1.777 ± 0.5007, *p* = 0.0216) and supraphysiological dose (8.620 ± 0.5910, *p* = 0.0005) compared to solvent control (1.787 ± 0.9884) (Figure 3). Perturbation of the inner mitochondrial polarity is an early event in apoptosis and characterises the initiation of the intrinsic apoptotic pathway. MOMP advances to depolarisation of the mitochondrial membrane, which permits the release of various proapoptotic proteins into the cytoplasm, such as Ca^2+^, cytochrome C, and APAF-1, which results in the aptosome formation, together with caspase-9, and the activation of effector caspases 3 and 7, which cause downstream apoptotic changes including phosphatidyl serine flipping and DNA damage [36]. Treatments with 250 nM and 2500 nM are therefore crucial for inducing the apoptotic point of no return in SiHa cervical cells.

#### 3.2.2. Assessment of Externalisation of Phosphatidylserine (PS) to the Outer Cell Membrane Surface Measured by an Annexin V assay

**Figure 4:**
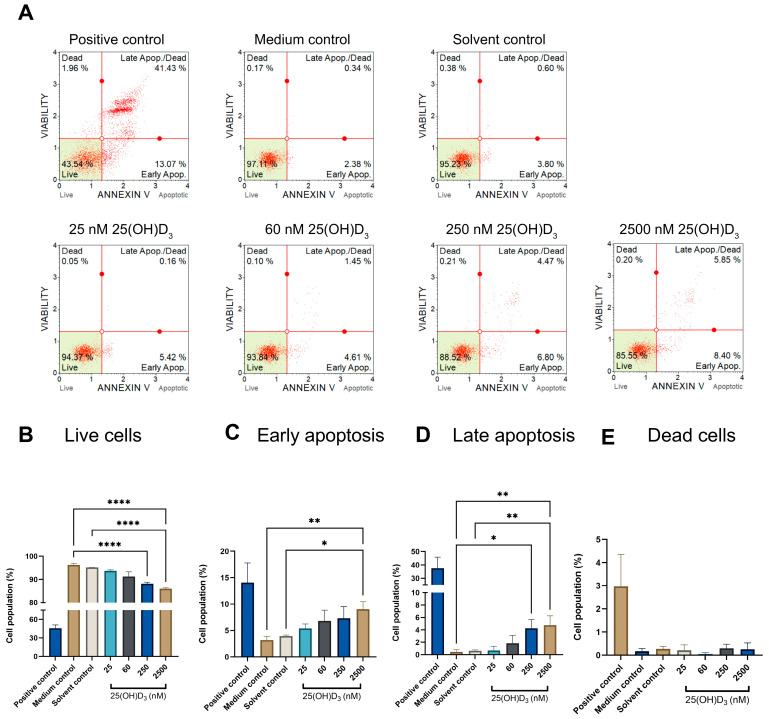
Identification of phosphatidylserine externalisation to the cell membrane’s surface was detected by flow cytometry using the annexin V assay. The dot plots of control and experimental SiHa cells (**A**), were analysed by bar graphs that differentiated the live cell population (**B**) from early apoptosis (**C**), late apoptosis (**D**), and dead cell (**E**) populations. The positive control was treated with actinomycin to a final concentration of 0.1 μg/mL. Data are expressed as mean (±SEM) from three independent experiments, each performed in triplicate (* *p* ≤ 0.05; ** *p* ≤ 0.01; **** *p* ≤ 0.0001).

Significant annexin V staining was detected in early and late stages of apoptosis (Figure 4), as especially evident in cell populations in the late apoptotic stage, at which point physiological treatment (4.253 ± 0.8012, *p* = 0.0173) and supraphysiological treatment (4.757 ± 0.8690, *p* = 0.0061) doses showed significance in comparison to the solvent control (0.6367 ± 0.07446). The detection of annexin V staining in the late apoptotic population indicates cells stained with the dead cell marker and annexin V. Thus, 25(OH)D3 induced a phosphatidyl serine flipped pattern at physiological and supraphysiological doses, which is a characteristic biochemical biomarker of apoptosis.

#### 3.2.3. Executioner Caspase Assay Detected Significant Increase in Caspase-3 and -7 Analysed by Muse™ Caspase-3/7 Assay at Physiological and Supraphysiological 25(OH)D3 Treatment Doses in SiHa Cells

**Figure 5:**
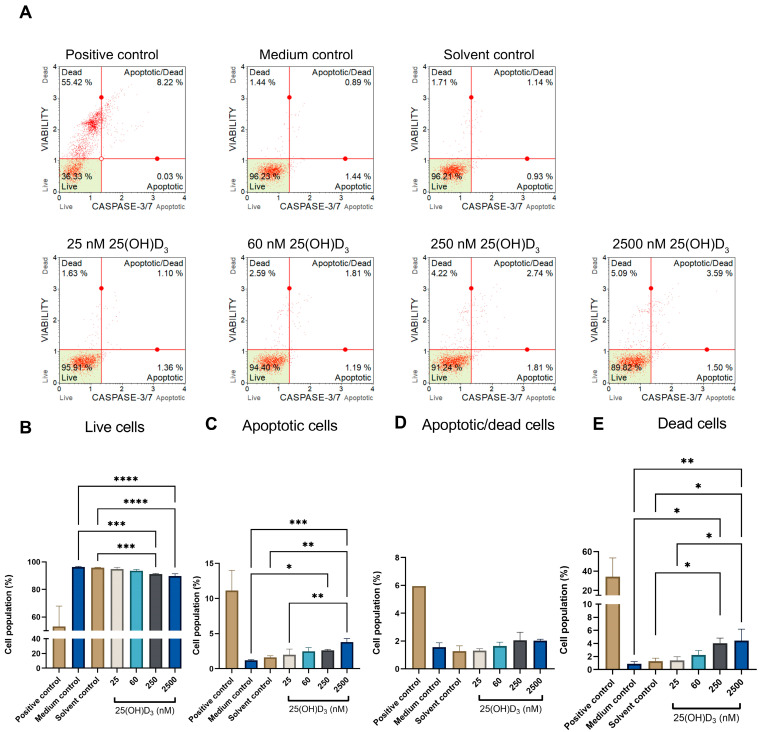
Flow cytometric analysis of effector caspases -3 and -7 in 25(OH)D3-treated SiHa cultures. Dot plot analysis (**A**) shows control and experimental cultures treated with 25(OH)D3, categorised into live, apoptotic, apoptotic/dead and dead populations based on dual staining by propidium iodide and caspases -3 and -7. The cell population categories were analysed and represented in bar graphs (**B**–**E)**: live (**B**), apoptotic (**C**), apoptotic/dead cells (**D**) and dead cells (**E**). The positive control was treated with actinomycin to a final concentration of 0.1 μg/mL. Data are expressed as mean (±SEM) from three independent experiments, each performed in triplicate (* *p* ≤ 0.05; ** *p* ≤ 0.01; *** *p* ≤ 0.001, **** *p* ≤ 0.0001).

Effector caspases -3 and -7 were significantly elevated in the apoptotic and dead cell populations in SiHa cells treated with physiological and supraphysiological 25(OH)D3 doses (Figure 5). The apoptotic population evaluated by the caspase3/7 assay showed an increase at a physiological dose (2.617 ± 0.0864, *p* = 0.0414) in comparison to the medium control (1.200 ± 0.0472), and the SiHa cells treated with 2500nM demonstrated an increase of 3.800 ± 0.2857 (*p* = 0.0013) in comparison to the solvent control (1.620 ± 0.1389). The number of dead cells that expressed caspases was also significantly elevated at the physiological dose (4.027 ± 0.4493, *p* = 0.0388) and supraphysiological dose (4.443 ± 0.9977, *p* = 0.0138) in comparison to the solvent control (1.287 ± 0.2646). These data confirm that 25(OH)D3 treatments at 250 nM and 2500 nM activate distal apoptotic biochemical biomarkers, which precede the terminal morphological features of apoptosis. 

#### 3.2.4. Ultrastructural Analysis of Experimental Cultures Showed Cardinal Features of Apoptosis in 250 nM and 2500 nM 25(OH)D3 Treatments in SiHa Cells, Confirming the Presence of Morphological Apoptosis

**Figure 6:**
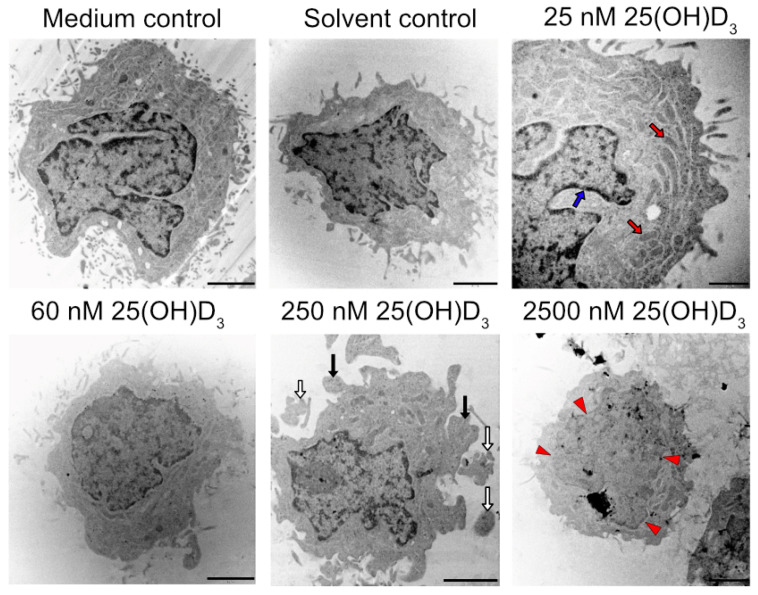
Ultrastructural analysis of SiHa control and experimental cultures analysed by TEM. Cell rounding and membrane blebbing (black arrows), apoptotic body formation (white arrows), and nuclear damage (karyolysis, red arrowheads). Red arrows indicate healthy mitochondria, and an intact nuclear membrane is indicated by blue arrows (scale bar = 1 μm).

Our study identified classical hallmarks of morphological apoptosis at physiological and supraphysiological 25(OH)D3 treatments. Cells were identified at various stages of apoptosis and demonstrated features of cell rounding, cell membrane budding (blebbing), nuclear damage (karyorrhexis and karyolysis), and apoptotic body formation. (Figure 6). Additionally, the cell membranes were intact, and cell swelling and features of autophagic-dependent cell death were absent. The morphological features of apoptosis support our findings of biochemical apoptosis, as evidenced by significant activation of early and late biochemical biomarkers at 250 nM and 2500 nM 25(OH)D3 treatments.

#### 3.2.5. Autophagy-Dependent Cell Death by LC3-II Detection and Necrotic Cell Death Assessed by LDH Were Not Significantly Increased in Experimental Cultures Spanning the 25(OH)D3 Treatment Range

**Figure 7:**
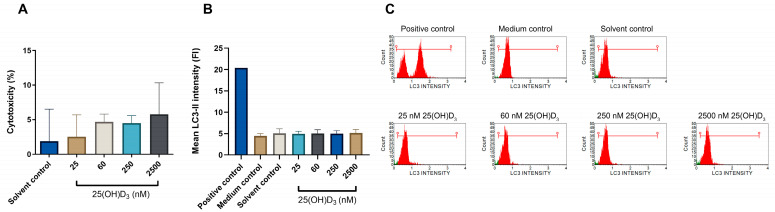
Evaluation of necrosis by the LDH release cytotoxicity assay, and measurement of autophagic-dependent cell death by LC3-II flow cytometric analysis in SiHa control and experimental cultures. Cytotoxicity was measured by the LDH release assay in control and experimental cultures (**A**). The y-axis identifies the percentage cytotoxicity, which indicates the activity of released intracellular LDH into the culture medium. Autophagic-dependent cell death by quantification of LC3-II is shown in bar graphs (**B**) and flow cytometric histograms (**C**) in the control and experimental SiHa cultures. Rapamycin (200 nM) was used to treat the positive control. All data are expressed as mean (±SEM) from three independent experiments, each performed in triplicate.

Cell necrosis (type three cell death) results in organelle swelling, lysis, the release of intracellular contents, and inflammation [50]. Therefore, necrosis is characterised by damaged and permeabilised cell membranes, which release intracellular cytosolic LDH into the growth medium of cell culture systems. LDH enzyme activity can be detected by the LDH release assay [40]. Experimental SiHa cultures did not show any significant change in LDH activity compared to control cultures (Figure 7A) and thus mitigated the induction of necrosis by 25(OH)D3. Furthermore, this finding suggests that the increased cell population in the sub-G1 phase observed in the cell cycle analysis was not a consequence of significant necrotic SiHa cell death at physiological and supraphysiological doses of 25(OH)D3.

Autophagy-dependent cell death, independent of apoptosis and necrosis, is measured by flow cytometric detection of lipidated LC3, which accurately characterises autophagy-dependent cell death [39]. There was no significant upregulation of LC3-II intensity in experimental SiHa cultures across the treatment range (Figure 7B,C). This suggests that 25(OH)D3 treatments neither induced autophagic-dependent cell death nor crosstalk between apoptosis and autophagy-dependent cell death.

### 3.3. Analysis of the VDMS Gene and Protein Expression in SiHa Control and Experimental Cultures

**Figure 8:**
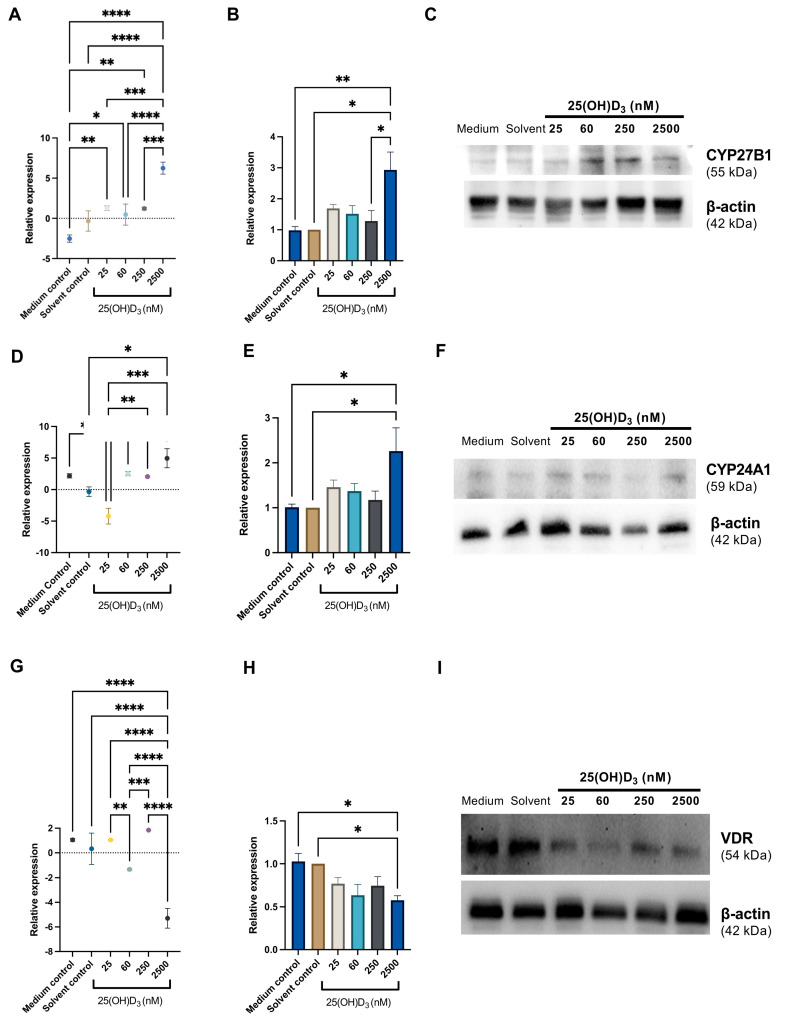
Gene and protein expression of CYP27B1, CYP24A1, and VDR in SiHa control and experimental cultures. Representative analyses of qPCR and Western blot for CYP27B1 (**A**–**C**), CYP24A1 (**D**–**F**), and VDR (**G**–**I**) are shown. qPCR data are presented as mean ± SEM for a representative of three independent experiments conducted in triplicate, and Western blot data are presented as mean ± SEM for three independent experiments. qPCR data (**A**,**D**,**G**) were analysed using the delta delta Ct method, and gene expression was normalised to 18 s housekeeper expression. Protein expression (**B**,**E**,**H**) was normalised to the β-actin loading control. Gene and protein expression were calculated as fold change relative to the solvent control. One-way ANOVA was used for data analysis, and *p* ≤ 0.05 was considered statistically significant (* *p* ≤ 0.05; ** *p* ≤ 0.01; *** *p* ≤ 0.001; **** *p* ≤ 0.0001).

CYP27B1 protein expression was significantly upregulated under supraphysiological treatment (2.933 ± 0.5749, *p* = 0.0103). The expression of CYP27B1 at the other treatment doses showed insignificant upregulation (25nM, 1.687 ± 0.1292; 60nM, 1.515 ± 0.2620; and 250 nM, 1.283 ± 0.3381) (Figure 8). These findings collectively show a trend of upregulation of CYP27B1 protein expression with 25(OH)D3 treatment dose, which is significant at the highest treatment dose. mRNA expression does not correspond to protein expression under physiological treatment, and this relationship suggests that mRNA is degraded or partially blocked in SiHa cells.

CYP24A1 mRNA expression was significantly increased under physiological and supraphysiological treatments; however, significant upregulation of CYP24A1 protein expression was observed under supraphysiological treatment (2.26 ± 0.5178, *p* = 0.027) (Figure 8). These data suggest that physiological 25(OH)D3-induced CYP24A1 mRNA levels do not uniformly correspond to protein synthesis levels, in contrast to supraphysiological treatment, under which gene and protein correlation for CYP24A1 was present.

The gene expression of VDR was significantly downregulated under supraphysiological treatment compared to all other treatments and controls. However, the VDR protein expression was significantly downregulated only under supraphysiological treatment compared to the medium control and solvent and control (0.5766 ± 0.05334, *p* = 0.0380). The other treatment doses showed a trend of insignificant depression of VDR protein expression (Figure 8).

## 4. Discussion

The autocrine and paracrine action of vitamin D precursor 25(OH)D3 and its fully activated form, 1,25(OH)D, mediate anticancer actions via intracellular mechanisms, particularly growth inhibition, cell cycle arrest, and apoptosis [15,51]. Our study demonstrates that 25(OH)D3 inhibits cell growth and cell viability, and induces apoptosis in SiHa cervical cells at physiological and supraphysiological doses via regulation of the autocrine VDMS by negative feedback in a dose-dependent manner.

Our cell viability data are similar to the dose-dependent inhibition observed under 100 nM, 200 nM, and 500 nM 1,25(OH)D3 treatments of HeLa S3 cervical cells [52] based on the enzymic conversion of a dye precursor by intracellular dehydrogenase activity using a colorimetric endpoint assay. This suggests that precursor 25(OH)D3 and activated 1,25(OH)D3 impair cell viability in cervical cancer cell lines.

Our study supports 25(OH)D3 induction of intrinsic apoptosis evidenced by biochemical and morphological features of apoptosis. Our observations are consistent with the Nomenclature Committee of Cell Death (NCCD) [36] definition of intrinsic apoptosis as an RCD initiated by extracellular or intracellular microenvironment perturbations, demarcated by MOMP, and affected by executioner caspases. Furthermore, an increase in the sub-G1 fraction of cells detected by cell cycle analysis shows apoptotic cells or cellular debris containing apoptotic bodies that comprise digested fragments of nuclear chromatin, which stain DNA poorly with the dead cell marker [49]. The degree of DNA fragmentation and lysis depends on the stage of apoptosis and is cell-specific [53]. In addition, studies investigating the anticancer effects of the prohormone cholecalciferol on cervical cancer cell lines, SiHa and CaSki, also show apoptotic induction at supraphysiological treatment dose [54,55].

A mechanism of apoptotic induction by cancer chemotherapeutics includes the upregulation of proapoptotic proteins compared to antiapoptotic proteins [36,56]. This is a crucial mechanism of vitamin D’s proapoptotic anticancer action [57]. A balance favouring pro-apoptotic protein synthesis and activation may mediate our observed finding of intrinsic apoptosis in SiHa 25(OH)D3-treated cells. Other regulatory molecular mechanisms contributing to the anticancer action of 1,25(OH)D3′s in cervical cancer cells include the downregulation of oncogenic voltage channels [52], upregulation of DDX5 RNA helicase action [58], and increased biogenesis of microRNAs regulating cancer pathways [59]. Collectively, these studies support the potential therapeutic application of precursor vitamin D metabolites (cholecalciferol, 25(OH)D3) and 1,25(OH)D3) in the clinical management of cervical cancer by targeting induction of apoptosis and other antitumorigenic mechanisms.

Our study further evidences an autocrine VDMS responsive to 25(OH)D3 in SiHa cervical cancer cells. Gene and protein expression studies of the VDMS suggest an intact negative feedback loop induced by 25(OH)D3 treatments in SiHa cells. The supraphysiological treatment induces concomitant mRNA and protein upregulation of 1-alpha-hydroxylase and 24-hydroxylase, and downregulation of VDR expression. In contrast, physiological treatment increases the 1-alpha hydroxylase mRNA transcript level, with no change in protein expression. The consistent downregulation of VDR across the treatment range suggests that signalling by liganded VDR is responsive to 25(OH)D3 treatment doses spanning insufficient (25 nM) to ten-fold supraphysiological (2500 nM) doses. The insufficient and deficient 25(OH)D3 treatments demonstrate insignificant changes in gene and protein expression of the vitamin D system; however, a trend of increasing activation and catabolism of 25(OH)D3 was observed. The negative inhibition of 25(OH)D3 treatments, downregulating VDR expression and inducing CYP24A1 in SiHa-treated cells, implies decreased intracellular signalling by VDR and increased catabolism by CYP24A1 to form partial-agonist or excretory vitamin D metabolites.

Two studies confirm the presence of VDMS in healthy and tumorigenic cervical tissue, as identified in our SiHa cell line model. However, the regulatory role and prognostic significance of VDMS are unclear and require further investigation [23,60].

The upregulation of CYP27B1 under physiological and supraphysiological 25(OH)D3 treatments observed in our study supports autocrine activation of the precursor 25(OH)D3 hormone to the fully activated potent 1,25(OH)D3. The expression of CYP27B1 in SiHa cells is consistent with another study that detected CYP27B1 RNA semi-quantitatively in healthy cervical tissue, HeLa cervical adenocarcinoma cells, and cervical tumours. There was no visible difference in the expression of 1-α-hydroxylase between normal cervical tissue (*n* = 4), cervical carcinoma (*n* = 8), and HeLa cells [61]. Another study investigated the effect of 1 nM and 100 nM cholecalciferol, 25(OH)D3, and 1,25(OH)D3 treatments in HeLa cervical cancer cells on VDMS mRNA transcript expression by real-time PCR. There was no significant change in 25-hydroxylase and 1α-hydroxylase expression; however, CYP24A1 showed significant upregulation [62]. In summary, CYP27B1 may regulate cell health by activating 25(OH)D3 precursor to 1,25(OH)D3 in healthy cervical tissue and cervical cancer. Therefore, precursors of vitamin D hormone may function as a chemotherapeutic in cervical cancer mediated by autocrine CYP27B1 activation.

The activated vitamin D metabolites transduce their signal via the VDR-RXR heterodimer complex to regulate VDRE-containing cell health genes [63]. Our study is consistent with other studies identifying VDR in cervical cancerous tissue. Reichrathe et al. [64] revealed that cervical carcinomas (*n* = 23) upregulated the immunoreactivity of VDR compared with normal cervical tissue (*n* = 15). Another study showed that although the mRNA transcripts of VDR were not increased in SiHa cells compared to normal cervical tissue, the VDR protein expression by immunohistochemistry was increased [60]. 

In summary, VDR expression is upregulated in cervical tumours; however, a negative feedback loop regulating autocrine vitamin D signalling via attenuated VDR expression, especially at a supraphysiological 25(OH)D3 dose, is likely to be operable in SiHa cells.

Studies investigating the action of fully activated 1,25(OH)D3 vitamin D metabolite show similar negative feedback regulation on 24-hydroxylase catabolism in cervical cancer cells, as evidenced in our study using 25(OH)D3 treatments. For example, Gonzales et al. [59] demonstrated that SiHa cells treated with doses of 0.01 μM, 0.1 μM, and 1 μM 1,25(OH)D3 upregulated CYP24A1 RNA expression, indicating an intact autocrine homeostatic negative feedback loop in response to the fully activated vitamin D metabolite treatments. Furthermore, Kloss et al. [62] also identified significant upregulation of 24-hydroxylase gene expression in response to 100nM 1,25(OH)D3 treatment in HeLa cells without changes in gene expression of 25-hydroxylase and 1-alpha hydroxylase. The 24-hydroxylase activity was also increased by 31.7-fold in a metastatic squamous cervical carcinoma (EC-50) by 10 nmol/L 1,25(OH)D3 treatment. However, this result was not associated with a change in cell growth assayed by incorporating radiolabelled thymidine into diethylstilbesterol and trypan blue assays [65]. In addition, evidence of negative feedback inhibition of CYP24A1 induced by autocrine 1,25(OH)D3 synthesis is supported by a transfected 1α-hydroxylase SiHa cell model treated with vitamin D metabolites [66] These preclinical studies collectively support the hypothesis of autocrine activation of 25(OH)D3 to 1,25(OH)D3 by 1α-hydroxylase in cervical cancer cells, which induces the CYP24A1 catabolic enzyme to attenuate an intracellular hypervitaminosis D environment. A putative role of CYP24A1 as an oncogene was proposed in [67], and a meta-analysis demonstrated that CYP24A1’s expression or single-nucleotide polymorphisms were correlated with cancer progression and drug resistance [68]. Preclinical investigations of autocrine CYP24A1 expression in cancer are similar to systemic regulation of vitamin D metabolism in humans, where upregulation of CYP24A1 prevents pathophysiological effects of hypervitaminosis D [16]. Thus, SiHa cervical cancer cells demonstrate a negative feedback autocrine VDMS, which optimally regulates cervical cell health. This observation will require a confirmatory investigation to identify vitamin D metabolites in 25(OH) D3-treated cervical cancer cells.

## 5. Conclusions

The treatment of SiHa cervical cells with 25(OH)D3 precursor hormone shows inhibited growth and apoptosis at physiological and supraphysiological treatment concentrations. The autocrine VDMS shows negative feedback under supraphysiological treatment, as evidenced by the upregulation of catabolic CYP24A1 and CYP27B1 and the downregulation of VDR gene and protein expression. Future studies can consider direct measurement of the vitamin D metabolome to confirm the regulation of the VDMS in SiHa cells. In addition, the 25(OH)D3 form of vitamin D precursor hormone can be considered a potential adjunctive chemotherapeutic in cervical cancer and invites exploration by clinical trials.

## Data Availability

The study data can be made available upon request to the corresponding author. The data presented in this study are available upon request from the corresponding author. The data are not publicly available due to the institutional restrictions regarding the current data submission and findings for degree purposes.
